# Alzheimer's disease detection using data fusion with a deep supervised encoder

**DOI:** 10.3389/frdem.2024.1332928

**Published:** 2024-02-12

**Authors:** Minh Trinh, Ryan Shahbaba, Craig Stark, Yueqi Ren

**Affiliations:** ^1^Department of Computer Science, University of California, Los Angeles, Los Angeles, CA, United States; ^2^Sage Hill School, Newport Beach, CA, United States; ^3^Department of Neurobiology and Behavior, University of California, Irvine, Irvine, CA, United States; ^4^Mathematical, Computational and Systems Biology, University of California, Irvine, Irvine, CA, United States; ^5^Medical Scientist Training Program, University of California, Irvine, Irvine, CA, United States

**Keywords:** Alzheimer's disease, multimodal fusion, dimensionality reduction, diagnosis prediction, Alzheimer's biomarkers, data integration, multiview data integration

## Abstract

Alzheimer's disease (AD) is affecting a growing number of individuals. As a result, there is a pressing need for accurate and early diagnosis methods. This study aims to achieve this goal by developing an optimal data analysis strategy to enhance computational diagnosis. Although various modalities of AD diagnostic data are collected, past research on computational methods of AD diagnosis has mainly focused on using single-modal inputs. We hypothesize that integrating, or “fusing,” various data modalities as inputs to prediction models could enhance diagnostic accuracy by offering a more comprehensive view of an individual's health profile. However, a potential challenge arises as this fusion of multiple modalities may result in significantly higher dimensional data. We hypothesize that employing suitable dimensionality reduction methods across heterogeneous modalities would not only help diagnosis models extract latent information but also enhance accuracy. Therefore, it is imperative to identify optimal strategies for both data fusion and dimensionality reduction. In this paper, we have conducted a comprehensive comparison of over 80 statistical machine learning methods, considering various classifiers, dimensionality reduction techniques, and data fusion strategies to assess our hypotheses. Specifically, we have explored three primary strategies: (1) Simple data fusion, which involves straightforward concatenation (fusion) of datasets before inputting them into a classifier; (2) Early data fusion, in which datasets are concatenated first, and then a dimensionality reduction technique is applied before feeding the resulting data into a classifier; and (3) Intermediate data fusion, in which dimensionality reduction methods are applied individually to each dataset before concatenating them to construct a classifier. For dimensionality reduction, we have explored several commonly-used techniques such as principal component analysis (PCA), autoencoder (AE), and LASSO. Additionally, we have implemented a new dimensionality-reduction method called the supervised encoder (SE), which involves slight modifications to standard deep neural networks. Our results show that SE substantially improves prediction accuracy compared to PCA, AE, and LASSO, especially in combination with intermediate fusion for multiclass diagnosis prediction.

## 1 Introduction

Alzheimer's disease (AD) is a progressive neurological disorder and the most common cause of dementia among older adults. The prevalence of AD is expected to surge to 13 million Americans by 2050, with millions more affected worldwide (Alzheimer's Association, 2023). With an increasing clinical load of older adults at risk of AD, one major bottleneck is accurate and timely diagnosis of AD using readily accessible data (Knopman et al., 2021), which has resulted in underdiagnosis of the condition (Connolly et al., 2011; De Levante Raphael, 2022). This is partly due to the severe shortfall of geriatricians and expert clinicians, who are critical to providing early diagnosis and care for older adults in the United States (Alzheimer's Association, 2023). As fluid biomarkers, including cerebrospinal fluid (CSF) and plasma assays, are being introduced for screening of AD (Jack et al., 2018), and with the recent draft of the “NIA-AA Revised Criteria for Diagnosis and Staging of Alzheimer's Disease” being made available (Andrews et al., 2023), the field of AD research has moved toward embracing more biologically-driven definitions of disease. Given the increased focus on AD biomarkers and the availability of large-scale datasets for AD research, the field needs to urgently understand how to best integrate distinct data modalities to assist in making early and accurate AD diagnosis.

Past work in the area of AD diagnosis using large-scale datasets and computational tools has been divided in their use of multimodal data. Some studies have primarily relied on single-modal inputs, commonly structural MRI. For instance, Lazli (2018) implemented a support vector machine enhanced with fuzzy c-means to perform binary classification (HC vs. AD-dementia) using only MRI images, achieving a 75% accuracy. Korolev (2017) compared several 3D convolutional neural networks (CNN) for classification of HC vs AD-dementia using only MRI scans, achieving the best accuracy with ResNet at 80%. While these studies showcase the potential of statistical machine learning methods in AD diagnosis, their scope is constrained by lack of data integration across modalities. Other studies have indicated that integrating diagnostic data across multiple modalities may enhance computational methods of AD detection by allowing for a more holistic understanding of an individual's health profile (Kohannim, 2010). Some of these studies have introduced various techniques to integrate or “fuse” multiple modalities (Suk et al., 2014). Many of these studies have focused on fusing neuroimaging modalities in prediction AD status, including using MRI and PET scans (Shi et al., 2017; Punjabi et al., 2019; Song et al., 2021).

More broadly, different fusion methodologies have been introduced in machine learning, with applications to multi-omics (Chaudhary et al., 2018) and medical outcomes prediction (Ding et al., 2022). Three main strategies for fusion have been explored, including early fusion, intermediate fusion, and late fusion (Stahlschmidt et al., 2022). These strategies can be viewed as combining data sources at the signal-level (early), feature-level (intermediate), or decision-level (late) across models (Meng et al., 2020). In the field of AD prediction, a few studies have explored each strategy (Qiu et al., 2018, 2022; Lee et al., 2019), with more focus placed upon early and intermediate fusion methods (Huang et al., 2020). Late fusion typically ignores interactions across modalities and relies heavily on decision-level differences between models trained on different modalities, which is a major drawback in AD prediction, as diagnosis is never made based upon considering each source of information in isolation. In intermediate fusion pipelines especially, applying dimensionality reduction (DR) for each data modality plays a central role in extracting useful features for prediction. With the rise of different fusion strategies, it remains unclear what the most optimal method of data integration is. Furthermore, among studies that apply data fusion in the area of AD research, no exploration has been made to systematically determine how well different DR methods perform in the context of data fusion.

In this study, we introduce a novel DR method, the supervised encoder (SE), that aims to optimize prediction performance in a data fusion pipeline for AD diagnosis prediction. We systematically compare a representative variety of existing DR methods, popularly used in the field, with commonly used data fusion strategies to determine how our SE method performs. We first hypothesize that applying multiple modalities of diagnostic data rather than single modalities would enable statistical machine learning models to predict AD diagnosis with higher accuracy. Secondly, and more importantly, we hypothesize that applying DR methods, particularly the SE, would enable predictions models to extract more useful latent information and improve accuracy. In the realm of AD diagnosis prediction, we still cannot confidently determine the optimal data integration method *a priori* when presented with multimodal data. We aim to provide a framework for answering this question in this study.

In predicting AD diagnosis, including cognitively unimpaired (healthy control, HC), mild cognitive impairment (MCI), and dementia due to AD, we focus on using clinical and structural MRI data modalities. While other neuroimaging modalities, including FDG-PET, have been widely used in research studies and are sensitive to important aspects of AD pathology (Knopman et al., 2021), we prioritized using modalities with high levels of data availability to both obtain a more inclusive dataset and reflect the data modalities accessible to older adults in the American healthcare system. More accessible data sources have been previously demonstrated to be highly useful for AD status prediction and progression forecasting (Ren et al., 2023). However, there has been limited exploration of optimal data fusion strategies outside of neuroimaging modalities, particularly concerning the prediction of clinical AD diagnosis. Investigating optimal fusion methods will lead to improved disease prediction and enhance the utility of diverse types of multimodal biomarkers for AD diagnosis (Kohannim, 2010).

The results of this study will provide a road map for designing improved strategies to predict AD by effectively integrating diverse sources of information. By understanding how to best integrate distinct types of biological and clinical data to predict AD status, our findings will enable other researchers to better develop multimodal data pipelines that leverage the most relevant information from each source of information to make informed, accurate diagnosis predictions. In light of fluid biomarkers that will be used for AD screening in the near future, these methodological advances will be crucial for determining how to best integrate novel biomarkers in future studies. Furthermore, these insights will offer a deeper and more comprehensive understanding of the multimodal factors associated with AD and contribute to our knowledge of relevant risk factors.

## 2 Materials and methods

### 2.1 NACC dataset and modalities

We obtained data from the National Alzheimer's Coordinating Center (NACC), which included information from older adults with or at risk of developing Alzheimer's (Beekly et al., 2004; Weintraub et al., 2009). The dataset comprised of three data modalities:

- Uniform Data Set (UDS): Contains clinical information, behavioral survey responses, neuropsychological testing results, and additional diagnostic information (Morris et al., 2006; Besser et al., 2018).
- Structural MRI: Includes preprocessed volumetric, cortical thickness, and white matter hyperintensity values from the IDeA Lab (Director: Charles DeCarli, MD; University of California, Davis) following protocols from the Alzheimer's Disease Neuroimaging Initiative.
- CSF biomarkers: Provides protein assay information from lumbar punctures, including Aβ42, P-tau181, and T-tau levels. Please see the Supplementary material for more information.

NACC standardizes data collected across multiple Alzheimer's Disease Research Centers (ADRCs), which are located at major academic and research institutions in the United States and have specific research and recruitment focuses that serve to advance dementia research. This study uses data from 46 ADRCs. Some ADRCs focus on recruiting under represented populations in dementia research to improve the generalizability of findings from this data. The dataset consists of UDS visits from September 2005 to March 2023. Each visit is associated with a clinical diagnosis of HC, MCI due to AD, or dementia due to AD. For cognitively impaired individuals, we selected for subjects with primary or contributing etiology of AD using clinican judgement. We also focused our analysis on subjects over the age of 65, which isolates the population of older adults most at risk of developing AD (Knopman et al., 2021). We subsequently conduct binary classification (HC vs. any level of impairment, MCI or dementia due to AD) and multiclass classification (HC vs. MCI vs. dementia) of the clinical diagnoses. The sample sizes for each modality are 19,381 for UDS, 1,653 for MRI, and 1,628 for CSF.

### 2.2 Data preprocessing

To handle missing data, we applied different approaches for each data modality. When subjects had multiple clinical visits, we selected the baseline visit for analysis. For the UDS modality, we excluded features with over 10 percent missing data and removed incomplete records. The remaining dataset contained 19,381 patient records, each with 90 clinical features. For the MRI modality, we dropped incomplete records, resulting in 1,628 subjects with scan records, each containing 157 imaging features. While we initially considered including the CSF modality for data integration, only a very small sample of subjects had all three modalities of data (UDS, MRI, CSF) collected. Because of this, we only conducted single-modal analyses on CSF data. All CSF modality related methods and results are reported in the Supplementary material.

To create models accepting multimodal input, we merged the UDS and MRI datasets, which included 1,419 subjects. Subjects included in the merged datasets had at least one UDS visit and an MRI scan within 18 months of that visit. Summary demographic information for subjects in the merged UDS and MRI dataset are presented in Table 1. We report comparisons of each feature in Table 1 across the diagnostic groups, with significant differences in age, sex, education level, and ethnicity, and the Mini-Mental State Exam (MMSE).

**Table 1:** Summary demographic information of the study subjects for each diagnosis category.

			**Diagnosis**			
**Variable**	***N***	**Overall**, ***N*** **= 1,419**^*a*^	**HC**, ***N*** **= 898**^*a*^	**MCI**, ***N*** **= 273**^*a*^	**AD**, ***N*** **= 248**^*a*^	***p*** **-value** ^*b*^
**Age**	1,419	72 (65, 79)	70 (64, 77)	75 (70, 81)	75 (69, 80)	< 0.001
**Sex**	1,419					< 0.001
Female		813 (57%)	592 (66%)	114 (42%)	107 (43%)	
Male		606 (43%)	306 (34%)	159 (58%)	141 (57%)	
**Education**	1,419	16 (13, 18)	16 (14, 18)	16 (12, 18)	16 (12, 18)	0.030
**Race**	1,419					0.254
White		1,192 (84%)	741 (83%)	232 (85%)	219 (88%)	
African-American		151 (11%)	106 (12%)	26 (9.5%)	19 (7.7%)	
Other		76 (5.4%)	51 (5.7%)	15 (5.5%)	10 (4.0%)	
**Ethnicity**	1,419					0.007
Non-Hispanic		1,295 (91%)	812 (90%)	262 (96%)	221 (89%)	
Hispanic		124 (8.7%)	86 (9.6%)	11 (4.0%)	27 (11%)	
**MMSE**	1,200	29.0 (27.0, 30.0)	30.0 (29.0, 30.0)	28.0 (26.0, 29.0)	24.0 (20.0, 26.0)	< 0.001
Unknown		219	160	30	29	

^a^Median (IQR) or frequency (%).

^b^Kruskal-Wallis rank sum test; Pearson's Chi-squared test.

### 2.3 Classifiers

We identified two prediction tasks to evaluate in our analysis: binary and multiclass classification. Binary classification between HC and cognitively impaired subjects (MCI or dementia due to AD) is a relatively easy task and serves as a benchmark for assessing data fusion strategies and the more difficult multiclass problem. For binary classification, we apply logistic regression, an interpretable and commonly used method. For the multiclass task, we apply a random forest (Breiman, 2001) and a feed-forward neural network to distinguish between HC, MCI, and dementia due to AD. In our analysis, we refer to models based on logistic regression as binary classification models, while models that utilize either Random Forest or Neural Networks are called multiclass classification models. Because multiclass classification is a more challenging task, we report performance of both random forest and neural network models to better understand the performance of the DR and fusion strategies of interest. All the analysis is coded in Python 3.10 and utilizes the scikit-learn package version 1.2.1 (Pedregosa et al., 2011).

### 2.4 Model comparison

The datasets were split into 75% training and 25% testing sets for model development and evaluation, respectively. To ensure that our reported findings were not due to having a specific train-test split, we repeated the training and testing split randomly 50 times and report the aggregated results on the test set. This allowed us to report the mean test set performance and 95% confidence intervals to evaluate if differences between various classification results reached statistical significance with *p* < 0.05. Additional details on the features included in the analyses can be found in the Supplementary material.

### 2.5 Data fusion strategies

In this study, we aim to find an optimum combination of data fusion and DR. Specifically, we have explored three primary strategies: (1) **Simple data fusion**, which involves straightforward concatenation of datasets before inputting them into a classifier; (2) **Early data fusion**, in which features are concatenated first, then a DR technique is applied before feeding the resulting data into a classifier; and (3) **Intermediate data fusion**, in which DR methods are applied individually to each data modality before concatenating them to construct a classifier. We did not include late data fusion due to its lack of popularity in the AD literature (Huang et al., 2020).

### 2.6 Dimensionality reduction techniques

Because of the large variety of data considered within multiple diagnostic modalities, DR techniques have become an essential component of data fusion pipelines. DR methods aim to reduce the feature space while retaining the most critical information. It does this by finding a lower-dimensional representation of the data that captures the essential information. This can be done by projecting the original data onto a lower-dimensional space, resulting in a reduced set of derived variables obtained from the original measurements. Typically, these new derived variables can capture most of the information present in the original data (Van Der Maaten et al., 2009). Past work on multimodal neuroimaging fusion has demonstrated the usefulness of DR, either as a separate step in the pipeline or as a part of the classifier used (Punjabi et al., 2019). Feature selection is a special type of DR that involves choosing a subset of the original features based on their relevance to the task at hand, effectively discarding less important variables (Zebari et al., 2020). By doing so, we retain the interpretability of the variables, which is advantageous in understanding feature importance for disease prediction.

For DR, we compare several unsupervised and supervised alternatives. More specifically, we use Principal Component Analysis (PCA, Hotelling, 1933), Least Absolute Shrinkage and Selection Operator (LASSO, Tibshirani, 1996), Denoising Autoencoder, (AE, Rumelhart et al., 1986; Kumar et al., 2014), and Supervised Encoder (SE, Shahbaba et al., 2022). These methods were chosen due to their popularity of use in the literature, representation of the different supervised and unsupervised methods, and varying levels of complexity in capturing nonlinear interactions across features.

#### 2.6.1 PCA

**Figure 1:**
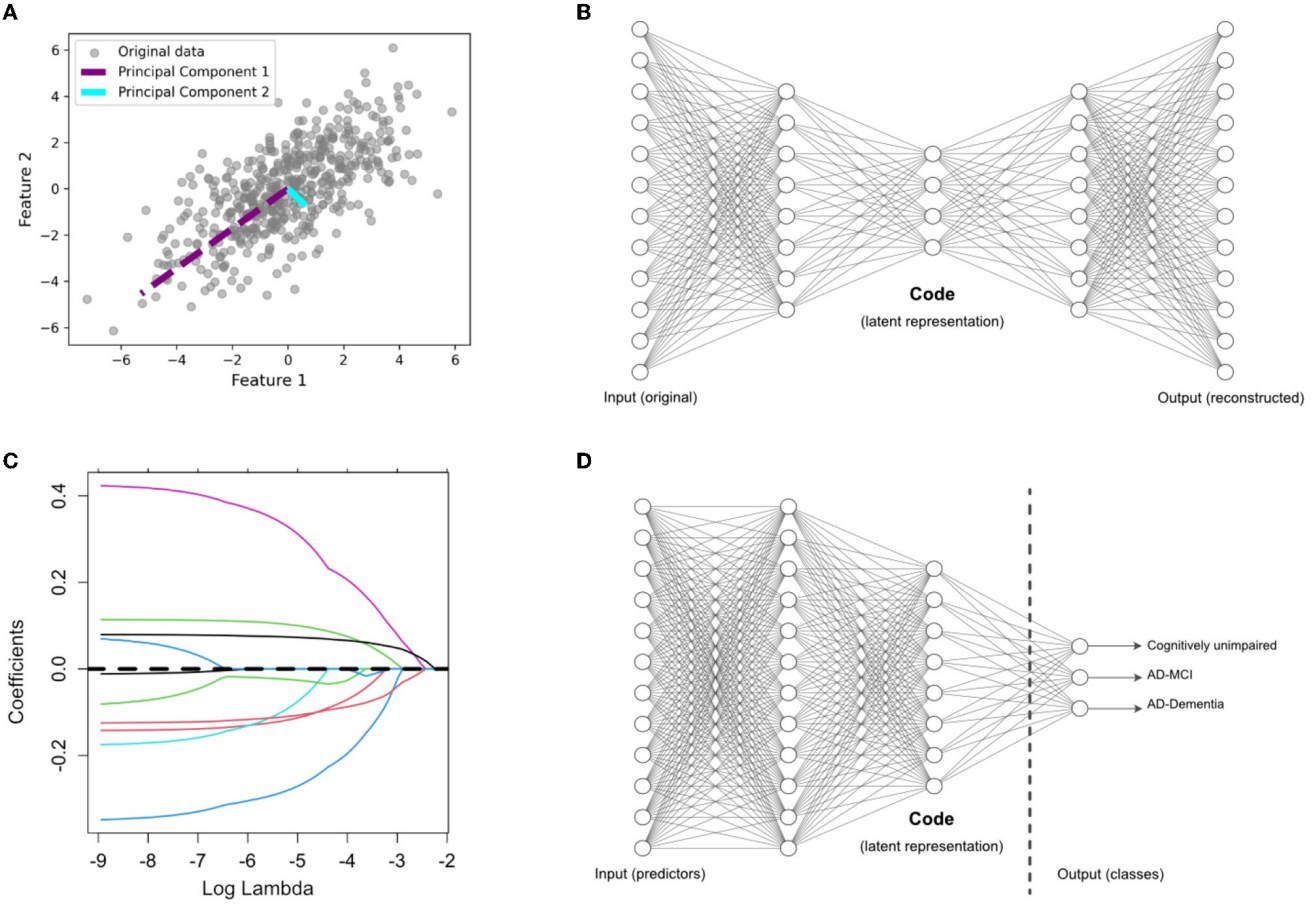
Overview of dimensionality reduction methods. **(A)** Principal component analysis (PCA), an unsupervised feature extraction method. **(B)** Autoencoder (AE) architecture for latent feature extraction. Conceptually, the AE can be interpreted as a nonlinear PCA. **(C)** LASSO, which applies a penalty term to identify the most useful features (with nonzero coefficients) in a supervised prediction problem. **(D)** Supervised encoder (SE) based on a deep neural network, with the latent features extracted from the bottleneck prior to the output layer. The SE extends the benefits of a supervised approach, like the LASSO, by finding more complex and nonlinear latent features.

This is a well-established linear and unsupervised DR technique widely employed in various domains. It aims to project high-dimensional data into a lower-dimensional subspace by accounting for maximum variance along orthogonal dimensions. This is achieved by finding orthogonal vectors called principal components (Hotelling, 1933). The first principal component explains the most variance in the data, the second one explains the second-most, and so on. This is shown visually in Figure 1A on a toy example of a two-dimensional feature space. Mathematically, the first principal component is obtained as follows:


(1) $$\begin{array}{l} \text{Maximize: Var}\left(Xw_{1}\right)\;\text{subject to }||w_{1}|{|}_{2}=1 \end{array}$$

In Equation 1, ***X*** represents the standardized input matrix, with a mean of zero and a variance of one. In our study, ***X*** comprises a set of *p* variables used for predicting AD. The vector *w*_1_ is the weight (also called loadings), and Var(***X**w*_1_) is the variance of the projected data (also called scores). The second principal component, *w*_2_, is orthogonal to *w*_1_ and provides scores, Var(***X**w*_2_), with the second highest variance. The subsequent principal components re obtained in a similar way to Equation 1 for maximizing the variance along each orthogonal dimension. After finding all the principal components, we limit our analysis to the first *q*≪*p* scores, ***X**w*_1_, ***X**w*_2_, …, ***X**w*_*q*_, which are treated as new derived variables capturing most of the information provided by the original variables. We chose the optimal number of components based on using scree plots to visualize the added value of each additional component. This value, often referred to as the “knee” of the plot, often included the top eight principal components for each pipeline.

#### 2.6.2 Denoising autoencoder

The AE (Rumelhart et al., 1986) is another unsupervised DR technique and can be considered as a nonlinear alternative to PCA. Figure 1B shows the architecture of a standard AE, which consists of two main components: an encoder and a decoder. The encoder compresses the input data into a lower-dimensional representation, often referred to as a bottleneck or latent space, while the decoder reconstructs the original input from this representation. Here, we use a variation of AE, called denoising AE (Kumar et al., 2014), which is designed for learning robust representations of data by introducing noise to the input and training the network to reconstruct the clean data. By training the AE to reconstruct the original, uncorrupted input from a corrupted version, it learns to capture the essential information while filtering out the noise. This makes denoising AEs particularly useful for extracting relevant features from noisy datasets, such as those encountered in medical research. This technique has been used in neuroimaging fusion and may be useful for clinical data fusion as well (see Shi et al., 2017). Here, we tuned the AE architecture in initial exploratory analyses on the training data and chose to implement to a five-layer AE (two layers for the encoder and decoder, respectively) with four nodes in the bottleneck layer. Adding more layers to the AE did not substantially change our final results. Details regarding the architecture of the AE are included the Supplementary material.

#### 2.6.3 LASSO

This is a linear regression technique used for feature selection through regularization to avoid overfitting (Tibshirani, 1996). LASSO adds a penalty term to the linear regression loss function, which encourages some regression coefficients to be exactly zero, effectively performing supervised DR. This is shown visually in Figure 1C on a toy example of a *p*-dimensional feature space in which some features have been dropped after the selection process, indicated by a coefficient of zero. LASSO aims to minimize the following objective function:


(2) $$\begin{array}{l} \text{Minimize:}\frac{1}{2n}||y-Xw|{|}_{2}^{2}+\lambda ||w|{|}_{1} \end{array}$$

where ***y*** is the target variable, ***X*** is the feature matrix, ***w*** is the coefficient vector, ||·||_2_ is the *L*2-norm, and ||·||_1_ is the *L*1-norm. In Equation 2, λ controls the strength of the penalty, influencing the degree of sparsity in the coefficients, which we tuned on our training data.

#### 2.6.4 Supervised encoder

As shown in Figure 1D, this is a modified version of the standard supervised feed-forward neural network designed for DR (Shahbaba et al., 2022). In this case, the last hidden layer (i.e., the last layer connected to the output layer) is designed as a bottleneck with a smaller number of nodes. After training the network in the usual way, the values of these nodes in the bottleneck are extracted as a low-dimensional representation of the original data. We hypothesize that this method, which has not been previously explored in AD prediction, may improve upon existing methods in multimodal fusion. We designed the SE architecture to be comparable to the AE architecture, with two layers prior to the code, which contained 10 nodes. This allowed us to compare the relative performance of the SE to the AE. Details regarding the architecture of the SE are included the Supplementary material.

### 2.7 Pipelines

**Figure 2:**
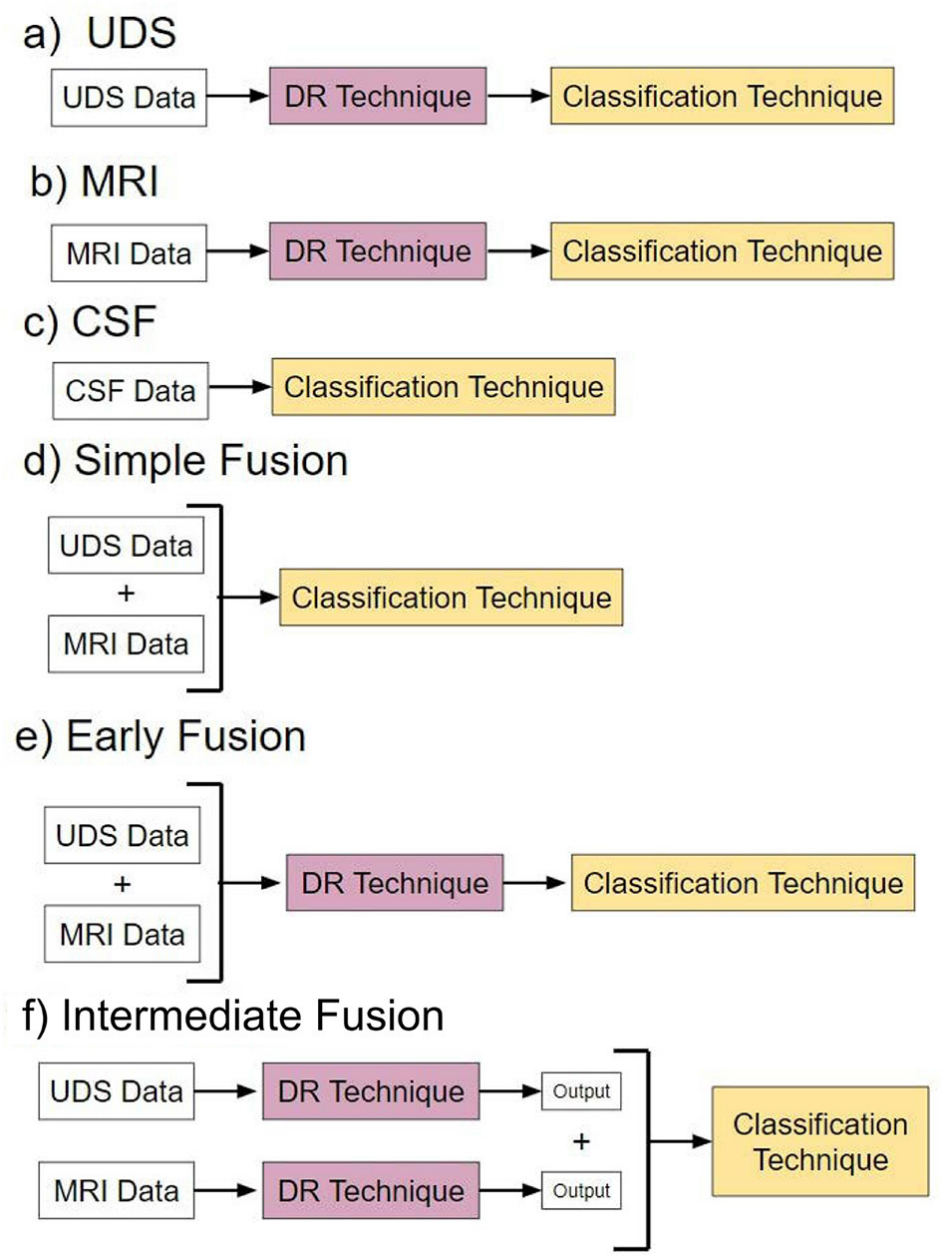
Pipelines. Pipelines **(a–c)** are models with different types of single-modal input. Pipelines **(d–f)** are models utilizing different forms of multimodal data fusion.

We evaluated six overarching analysis pipelines, each utilizing a different type modality input, in which a specific fusion method was applied if the pipelines included multimodal data (Figure 2). Pipelines a-c are “baseline models” that use single-modal input. Pipelines d-f are “fusion” models that use multimodal input consisting of both UDS and MRI data.

Pipeline a, named “UDS,” involves models using UDS input. We designed 12 such models. The 12 models are derived from the possible combinations of the four DR methods and the three classification methods. Four of the 12 models are binary, i.e., based on logistic regression. Eight models are multiclass, using either random forest or neural network to perform diagnosis.

Pipeline b, named “MRI,” consists of models using MRI input. Similar to pipeline, we designed 12 models.

Pipeline c, named “CSF,” involves models using CSF input. Due to the low dimensionality of the dataset (3 features), no DR was performed. See Supplementary material for more information.

Pipeline d, named “Simple Data Fusion,” consists of models designed to apply multimodal input to one of the three classification strategies without DR. Thus, there were three models designed for this case.

Pipeline e, named “Early Data Fusion,” consists of models designed to use multimodal input concatenated prior to use in a composite model with a specific DR technique and a classifier. Similar to pipeline a, there are 12 models created for pipeline e.

Pipeline f, named “Intermediate Data Fusion,” consists of models designed to apply a DR technique individually to UDS and MRI before passing the resulting dataset to a classifier. As before, there were 12 models for pipeline f.

### 2.8 Voting ensemble models

To perform comparisons between different pipelines each consisting of multiple models, we have created voting ensemble models. These models produce an output from the “majority vote” of multiple models. Two ensemble models were created per pipeline: one for binary classification models, and one for multiclass classification models. Thus, a total of 12 ensemble models were created. We compare the performance of these ensemble models with the individual models across all pipelines to determine the optimal DR and fusion strategy.

## 3 Results

Models using a single modality of data achieved the best classification results when using SE or PCA for DF. Table 2 shows the results obtained when various DR methods are applied to classifiers built using a single modality. We compared different models by assessing if there was any overlap of the 95% confidence intervals of accuracy rates evaluated on the test set data, with non-overlapping intervals indicating significantly different classification performance at a p-value of 0.05. The SE method provides the best overall results for UDS data processing, significantly outperforming other methods. Of note, LASSO performed similarly to SE using the neural network classifier (*p* ≥ 0.05). For MRI feature processing, PCA performed the best overall, but its performance was often on par with that of models using LASSO and SE, as indicated by overlapping confidence intervals. Models using AE as the DR method often resulted in the lowest accuracy value compared to results from models with other DR methods. Note that no DR method was used for the CSF data since there were only three variables in the dataset (see Supplementary material).

**Table 2:** Baseline models with single-modal input.

**UDS**			
	Binary	Multiclass	
	Logistic reg.	Random forest	Neural network
PCA	0.843 (0.841, 0.844)	0.780 (0.778, 0.781)	0.784 (0.783, 0.786)
AE	0.754 (0.744, 0.763)	0.700 (0.688, 0.711)	0.731 (0.722, 0.740)
LASSO	0.862 (0.861, 0.864)	0.804 (0.802, 0.805)	0.806 (0.805, 0.808)
SE	**0.880** (0.879, 0.882)	**0.810** (0.808, 0.811)	**0.809** (0.808, 0.811)
**MRI**			
	Binary	Multiclass	
	Logistic reg.	Random forest	Neural network
PCA	**0.793** (0.788, 0.797)	0.689 (0.682, 0.695)	**0.704** (0.698, 0.709)
AE	0.700 (0.691, 0.710)	0.620 (0.612, 0.628)	0.652 (0.645, 0.658)
LASSO	0.785 (0.780, 0.791)	**0.691** (0.686, 0.697)	0.698 (0.692, 0.704)
SE	0.781 (0.776, 0.786)	0.683 (0.677, 0.688)	0.673 (0.666, 0.679)

Test set classification accuracy values are reported for baseline models with single-modal input using various DR techniques. Mean values (95% confidence interval) are from 50 random testing splits. Bold values indicate the best performing DR method for each classifier.

For multimodal datasets, we observed that models using SE typically ranked as either the top model or in second place compared to other DR methods. The SE models performed significantly better than all other models using different DR methods in the intermediate fusion pipeline and outperformed all other models except for LASSO in the early fusion pipeline (*p* < 0.05). Pipelines using LASSO for early fusion performed significantly better than those with SE (*p* < 0.05), but this difference in performance was relatively modest. Overall, SE-based models achieved the highest classification performance and significantly outperformed other methods (*p* < 0.05, Table 3).

**Table 3:** Comparison of fusion methods.

**Simple data fusion**			
	Binary	Multiclass	
	Logistic reg.	Random forest	Neural network
No DR	0.799 (0.791, 0.807)	0.831 (0.825, 0.837)	0.795 (0.789, 0.800)
**Early data fusion**			
	Binary	Multiclass	
	Logistic reg.	Random forest	Neural network
PCA	0.850 (0.846, 0.855)	0.767 (0.761, 0.772)	0.782 (0.777, 0.787)
AE	0.798 (0.792, 0.805)	0.737 (0.731, 0.743)	0.760 (0.754, 0.766)
LASSO	**0.876** (0.872, 0.881)	**0.834** (0.828, 0.839)	**0.818** (0.813, 0.824)
SE	0.863 (0.858, 0.868)	0.794 (0.787, 0.800)	0.786 (0.779, 0.794)
**Intermediate data fusion**			
	Binary	Multiclass	
	Logistic reg.	Random forest	Neural network
PCA	0.871 (0.867, 0.875)	0.803 (0.797, 0.808)	0.800 (0.795, 0.805)
AE	0.760 (0.750, 0.769)	0.729 (0.718, 0.739)	0.721 (0.712, 0.731)
LASSO	0.877 (0.873, 0.882)	0.819 (0.813, 0.824)	0.809 (0.803, 0.814)
SE	**0.916** (0.912, 0.920)	**0.864** (0.861, 0.868)	**0.867** (0.862, 0.872)

Test set accuracy values are reported for each different fusion strategy using multimodal inputs from UDS and MRI data. Mean values (95% confidence interval) are from 50 random testing splits. Bold values indicate the best performing DR method for each classifier.

**Figure 3:**
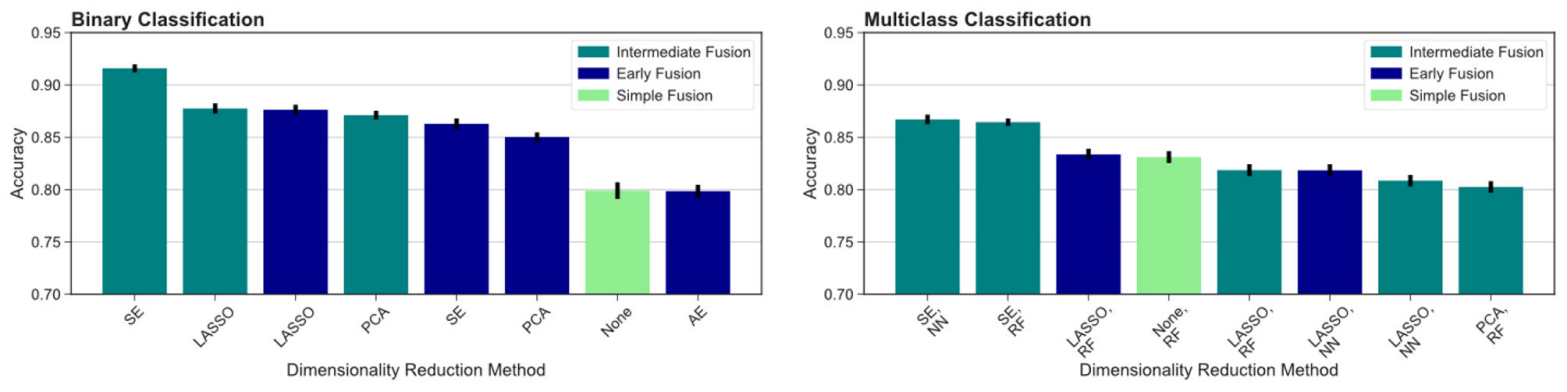
Comparing different fusion methods. We compare the top ranked fusion pipelines for binary **(left)** and multiclass **(right)** classification across DR methods. Intermediate fusion with SE consistently ranked as the top model in both classification tasks. All binary classification tasks used logistic regression as the classifier, and the multiclass models are labeled with the classifier used (RF, random forest; NN, neural network).

We then compared different data fusion strategies using various DR techniques. As indicated by the top ranked models in Figure 3, the best results were obtained using the intermediate fusion strategy in combination with the SE method (*left*: binary classification, *right*: multiclass classification). The complete results are reported in Table 3. For binary classification, this combination provides 91.6% accuracy rate. The corresponding accuracy rate for the multiclass task is 86.7% using a neural network classifier and 86.4% using a random forest classifier. The drop in performance when comparing intermediate fusion with SE to the next best model is significant and supports the notion that this pipeline is the most optimal data integration method (*p* < 0.05). Additionally, the difference in performance between the top model using SE and the second ranking models using other DR methods for the intermediate fusion pipeline is much greater than the gaps between top and second ranking models across DR methods for other fusion pipelines. This indicates that the high level of accuracy using intermediate fusion with SE is a more substantial improvement in performance compared to other fusion and DR methods.

To further test our hypotheses about the advantage of multimodal input in AD prediction, we also compared model accuracy rates from UDS and MRI pipelines against accuracy rates from pipelines involving data fusion using the Voting Ensemble approach (Table 4). As indicated by the classification performance, not every data fusion strategy is superior to single-modal models. However, the top ranked model always comes from a multimodal pipeline with an appropriate data fusion strategy. Intermediate fusion remains the best approach when utilizing an ensemble of models pipeline, replicating our findings from comparing different fusion methods. Overall, intermediate fusion ensemble models performed the best out of all pipelines, achieving 89.5% binary classification accuracy and 84.1% multiclass classification accuracy. We observe that the simple data fusion pipeline's multiclass ensemble model achieves an similar accuracy of 81.6% as the early fusion's multiclass model (*p* ≥ 0.05). However, the early fusion's single models' and ensemble model's results are much better than the results from the simple data fusion pipeline (Table 3). Intermediate fusion ensemble models significantly outperform simple data fusion's binary classification model (Table 2) and multiclass ensemble model (Table 4).

**Table 4:** Comparison of ensemble models.

**Voting ensemble**		
**Modality**	**Binary**	**Multiclass**
UDS	0.868 (0.866, 0.869)	0.812 (0.810, 0.814)
MRI	0.800 (0.795, 0.805)	0.713 (0.707, 0.719)
Simple data fusion	—	0.816 (0.811, 0.821)
Early data fusion	0.882 (0.878, 0.887)	0.816 (0.811, 0.822)
Intermediate data fusion	**0.895** (0.891, 0.899)	**0.841** (0.835, 0.846)

12 ensemble models were created, 2 per pipeline. The multiclass ensemble model produced diagnoses based on a majority vote of all multiclass models within that pipeline. The binary ensemble model performed similarly, combining all binary models that applied different DR methods in the pipeline. The simple fusion pipeline for binary classification only included one model, which did not qualify it for building an ensemble model. Mean values (95% confidence interval) are from 50 random testing splits. Bold values indicate the best performing fusion strategy for each classifier.

**Figure 4:**
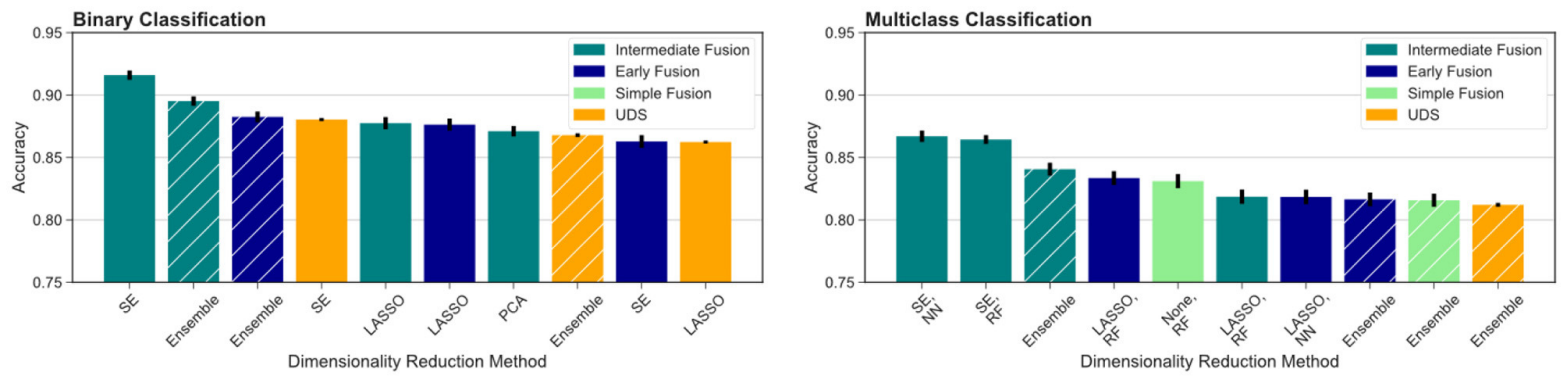
Comparison across all models evaluated. We report the top ten models for binary **(left)** and multiclass **(right)** classification across fusion and DR methods. Ensemble models results are indicated by hashed bars, and the remaining results are from single-model pipelines. Intermediate fusion with SE consistently ranked as the top model. Models using single-modal CSF data did not rank among the top 10 models for either classification task. All binary classification tasks used logistic regression as the classifier, and the multiclass models are labeled with the classifier used (RF, random forest; NN, neural network).

To compare across all the pipelines presented, we ranked the highest ten classification accuracy rates achieved by any model in the study. For the binary classification task, which distinguishes between cognitively unimpaired and impaired individuals (MCI or dementia), we observed that multimodal models performed the best, including the top three models and seven out of the top ten models overall (Figure 4, left). This was especially evident for the more difficult multiclass classification task (HC vs. MCI vs. dementia). Here, the top nine ranking models out of the top ten models overall for multiclass classification all used multimodal inputs (Figure 4, right). Focusing on the binary classification tasks, we observed that the drop in accuracy values after the top ranked model is much more substantial, indicating that the use of intermediate fusion helps boost performance noticeably. In contrast, the most prominent finding for the multiclass results is the high performance of intermediate fusion models utilize SE, which was the methodology employed by the top three ranked models.

## 4 Discussion

Our results support the hypothesis that combining modalities as input to statistical models can yield substantially higher accuracy using the right fusion strategy. Additionally, our results support the hypothesis that proper use of DR can lead to optimal data fusion strategies for predicting AD diagnoses. Most notably, multimodal models utilizing the SE to perform intermediate fusion achieved the highest accuracy rates, significantly outperforming all other methods (*p* < 0.05). Models built with the SE-transformed features achieve 91.6% accuracy with binary logistic regression, 86.4% accuracy in multiclass random forest, and 86.7% accuracy with multiclass neural network. These results also outperform existing deep learning pipelines for AD prediction that integrate MRI and clinical modalities from NACC data using alternative strategies (Qiu et al., 2020), strongly suggesting that applying the SE to perform intermediate fusion may be an optimal data fusion strategy for AD prediction. The next best single-modal input models, built with UDS, achieved 88.0% for binary classification and only 81.0% for multiclass classification. This indicates the strength of implementing intermediate fusion with SE, especially for more challenging multiclass prediction tasks. These accuracy values were higher than those of models built with single-modal MRI and CSF input features (see Supplementary material).

In comparing single modality and multimodal dataset results, it is clear that combining multiple sources of information does not always lead to better results. Thus, it is especially important to select the appropriate feature processing pipeline (considering DR and fusion methods) to achieve the optimal classification performance using multimodal data. For example, using just the UDS data could lead to accuracy rates as high as 88.0% for the binary classification task and 81.0% for the multiclass task. While using an appropriate fusion strategy (i.e., intermediate fusion with SE) could increase these accuracy rates to 91.6% and 86.7% respectively, less optimal strategies such as simple fusion could lead to substantially worse results. This may be due to the fact that sometimes a new source of information (such as MRI) might introduce more noise to the data and mask informative signals provided by the existing data (in this case UDS).

We observed that intermediate fusion significantly outperformed early fusion in many of the pipelines across our experiments. Compared to early fusion, the intermediate fusion ensemble models achieved significantly higher accuracy values in multiclass and binary classification tasks when utilizing SE and PCA methods (*p* < 0.05). The difference does not reach significance for most models using LASSO and some models using AE (*p* ≥ 0.05). The most prominent boost in performance was observed in using SE with intermediate fusion. This suggests that combining multimodal data later on (i.e., after DR) is more advantageous than combining earlier in the classification pipeline when utilizing the SE method. We reason that this is because intermediate fusion combines various sources of information after removing noisy information from each modality first. In doing so, this effectively strengthens useful signals from each data source, avoids masking diagnostically meaningful information due to noise introduced from other (possibly weaker) data sources, and allows the classifier to better learn the underlying patterns from each input modality. These results support the notion that future work in utilizing multimodal data for AD prediction should strongly consider applying intermediate fusion or similar strategies for data integration to obtain better prediction results. However, as evident in the performance of models using AE for feature extraction, simply applying a single DR method with a fusion strategy does not always guarantee an improvement in performance compared to simple fusion. This is likely due to the unsupervised nature of AE, which compresses features into a latent representation that can be prone to include noisy data. This then makes the prediction task more challenging, which explains the observed drop in performance.

More broadly, we found that applying DR methods, regardless of fusion methodology, yielded more accurate prediction results. We observed that none of the simple fusion classification pipelines, which do not implement DR, yielded higher prediction accuracy values than the best performing single-modal models, using UDS data only, that implement DR methods in their pipeline (Tables 2, 3). Additionally, a vast majority of the multimodal models that apply DR methods, especially with a intermediate fusion strategy, perform better than the simple fusion models, which do not use DR methods. Specifically, six binary classification models with DR significantly outperformed simple data fusion (*p* < 0.05) and four multiclass classification models with DR significantly outperformed simple data fusion (*p* < 0.05). The latter models were heavily driven by supervised DR methods, indicating that for the UDS and MRI feature space, using a supervised DR method can significantly boost prediction performance. In comparing early fusion to intermediate fusion, we observed that six intermediate fusion models performed better than their early fusion counterparts and only three early fusion models performed better than their intermediate fusion counterparts across all classification pipelines (*p* < 0.05). This supports our hypothesis that multimodal input models generally perform better when we apply DR methods and intermediate fusion tends to achieve better classification performance than the other fusion strategies.

The benefit of applying DR methods has been previously reported across different fields, including in predicting drug-target interactions and cancer status (Quinlan et al., 2015; Ezzat et al., 2017; Kabir et al., 2023). While combining DR methods with various classification models do not guarantee improved prediction performance, the vast majority of past work support the exploration of DR methods in the modeling pipeline to determine if specific DR methods may improve predictive accuracy. In the realm of AD prediction, Shi et al. (2017) have shown that using a denoising AE with neuroimaging data has the potential to greatly improve predictive performance. Using clinical data, Ren et al. (2023) have also shown that DR methods, including PCA and LASSO, are useful for identifying relevant, important features for improving classification performance. We extend upon those findings in this study by including both clinical and survey data in addition to neuroimaging features. We also compared a variety of other DR methods to determine the optimal method for predicting AD status using a large cohort from NACC. To our knowledge, this is the first work to systematically compare data integration and DR methodology using NACC data on such a large scale, with over a sample size of over 19,000 from the UDS modality.

For a majority of the tasks we examined, both binary and multiclass, the best performing DR method was the SE. Unsurprisingly, since the SE optimizes latent representations of the input features that optimize for classification performance, using SE-transformed features based on the training set tended to yield significantly better classification results on the test set. However, when we examine results from early fusion (Table 3), we observed that LASSO was the best performing DR method across all classification tasks and classifiers. We hypothesize that this may be due to the inherent differences between early fusion and other fusion methods. Because all features are concatenated prior to DR in early fusion, the DR method has a greater burden to identify relevant features that contribute most to optimal prediction. When constructing the SE model, the number of nodes in the code (latent representation) of the models inherently limits the dimensionality of the information used for subsequent classification. While this is beneficial in intermediate fusion, because each modality is considered separately to maximize signal to noise of the input features, this bottleneck becomes more limiting in early fusion due to potentially confounding signal and noise across modalities when learning the latent representations. As a result, the DR method has less capability to discern useful features from each modality and instead becomes a bottleneck for passing useful information to the classifier. On the other hand, LASSO has the strengths of being able to penalize the model from selecting features that do not carry additionally useful information for AD prediction, making it a powerful tool for removing noisy features regardless of the modalities considered.

We also observed that PCA was competitive with LASSO and SE for single modality models using MRI data. Due to the inherent noise introduced by the correlated and interrelated volumetric and cortical thickness values derived from a T1-weighted MRI scan used for these features, we hypothesize that PCA provides a more stringent method for distinguishing the top features due to its orthogonality requirements. This can serve to address the high levels of multicollinearity amongst features that make the high dimensional feature space difficult to navigate when attempting to reduce the dimensionality of the input features. Given this, we also observe that LASSO performs similarly to PCA (overlapping confidence intervals) for MRI-only models, due to its shared ability to penalize the model to reduce multicollinearity. In contrast, while the SE is able to find a compressed representation of the feature space, it does not employ the same penalization strategy of LASSO or orthogonal constraints of PCA to achieve the the same results. Thus, the SE lags slightly behind for the binary classification task and when using an neural network for multiclass classification with MRI-only models.

Using our model, we inferred the most significant risk factors of AD from features selected from the top performing intermediate fusion SE model using a neural network. We used permutation feature importance to obtain the top ranked UDS and MRI features from the model:

- **UDS:** TAXES, STOVE, REMDATES, TRAVEL, MEMPROB, APASEV
- **MRI:** HIPPOVOL, THIRVENT, LENTM, LINFTEMM, LROSMFM, LSUPFRM, RCMF, RENTM, RPARCENM, RPERCAL

The selected UDS features chiefly involve behavioral symptoms, such as having difficulty with performing everyday tasks. These include managing taxes (TAXES), using a stove or heating appliance for preparing drinks or cooking (STOVE), traveling in or out of the neighborhood (TRAVEL), and remembering key events (REMDATES). Other important UDS features include subsets of the Geriatric Depression Scale (self report of having more memory problems than most, MEMPROB) and the Neuropsychiatric Inventory Questionnaire (reports of apathy or indifference, APASEV). These important features corroborate our understanding of the interplay between functional and behavioral changes and neuropsychiatric symptoms along the Alzheimer's continuum (Masters et al., 2015; You et al., 2015). However, due to the limited range and specificity of the clinical diagnosis for capturing all potential causes of cognitive and behavioral changes, we should also note that changes in these features are not unique to AD pathology (Ismail et al., 2016).

Among the selected MRI features, the hippocampus (HIPPOVOL) and ventricular (THIRVENT) volumes appear to be most significant, which aligns with existing literature (Deweer et al., 1995; Devanand et al., 2007). Furthermore, left brain volumes seem to be of greater importance than right brain as they were ranked higher in feature importance according to our model. These left brain features included the thickness of the inferior temporal (LINFTEMM), rostral middle frontal (LROSMFM), and superior frontal cortex (LSUPFRM). Some of these regions have been previously identified in being significantly different between individuals with MCI and with dementia (Singh et al., 2006). The significant right brain features include the caudal middle frontal gray matter volume (RCMF) and thickness of the paracentral cortex (RPARCENM) and pericalcarine cortex gray matter volume (RPERCAL). Many of these regional cortical thickness values have been previously implicated in successful aging (Dominguez et al., 2021). Additionally, other top features include entorhinal cortical thickness values (LENTM, RENTM), which have been heavily studied in AD-related changes and implicated as one of the early markers of decline (Killiany et al., 2002; Velayudhan et al., 2013). These findings suggest that behavior and left brain features should be of particular importance when analyzing features that are useful for AD diagnosis prediction. However, interpreting these feature importance values must be done in the context of the NACC dataset used in this study and more work must be conducted to assess if the intermediate SE pipeline focuses on similar features in other datasets.

We also further analyzed if there were significant differences in model performance across each demographic group highlighted in Table 1. Demographic trends indicate that, in our dataset, the subjects with cognitive impairment are, on average, older and tend to be male and of Hispanic ethnicity. Using the results from the top model, with intermediate fusion, SE, and a neural network classifier, we computed the number of corrected predicted subjects in the test set stratified by demographic feature category. We conducted Chi-squared tests for each demographic variable, including age (grouped by decade), sex, education level (grouped as 0–12, 12–16, and 16 or more years of education), race, and ethnicity, and found no significant differences in prediction results across each feature (*p* ≥ 0.05). This indicates that our findings are not driven by demographic differences in the dataset or biased to the majority group within each demographic feature.

Our assessment of multimodal model performance has the limitation of being unable to consider CSF data in the multimodal model. Firstly, DR algorithms often did not select CSF features as significant in a pool of UDS, MRI, and CSF features. Secondly, the number of patients who have gone through both a UDS assessment and MRI screening within a reasonable timeframe, in addition to having CSF data collection, was too low to create a dataset for evaluating multimodal models that included CSF data. In the future, we may attempt to resolve these two issues by using other databases or by weighing the CSF features more heavily in the DR procedure. Our analyses were also limited by access to other key modalities in the AD field, such as PET scans and plasma biomarkers, which would greatly strengthen future work in this area. Plasma biomarkers, in particular, should be incorporated into future work investigating multimodal fusion in order to improve screening and early diagnosis using widely accessible data modalities. With additional modalities, more DR methods should also be explored in future studies, such as independent component analysis and linear discriminant analysis, to determine if certain DR methods are preferentially useful for particular data sources. We should also note that the target labels used to train the models come from clinical diagnosis, which may not always align with the underlying neuropathology observed postmortem (Beach et al., 2012). Due to this, the prediction accuracy results should be considered only in the context of predicting clinical judgments. Future work should also include comparisons with late fusion, which combines decision-level results from classifiers trained on different data modalities, as an additional strategy, which was not explored here due to its relative lack of use in the AD prediction literature. Additionally, it is difficult to generalize the results of our study to all older adults at risk of AD. The data is not representative of the broader American population due to its reliance on voluntary participation and a skewed demographic profile, with predominant representation of White individuals and patients with high levels of education. Future work in this area should seek to incorporate more diverse data sources and samples.

The results from this study demonstrate that computational models for AD diagnosis can be substantially enhanced with appropriate data fusion and DR methods. From our systematic comparison of various pipelines to combine different forms of diagnostic data, including clinical information and MRI scans, we present a comprehensive look at optimal multimodal data integration methods for AD prediction. This work serves as a foundation for designing improved computational strategies to predict AD by effectively integrating multimodal information. Due to limited exploration in the literature on optimal data integration methods that extend beyond neuroimaging modalities, these results are useful to many researchers in the AD field. DR plays a significant role in increasing accuracy of disease prediction tasks, particularly in pipelines using the SE method. This has the largest impact on improving accuracy results when applied in a multimodal data setting and for multiclass prediction, which are the more challenging and meaningful data integration tasks. We recommend researchers to explore intermediate fusion using various DR methods, including the SE we present here. Our findings on the effectiveness of using multimodal input, the best performing methods of DR, and the optimal data fusion strategy offer valuable insights in the development of more robust computational methods for diagnosing Alzheimer's.

## Data availability statement

Publicly available datasets were analyzed in this study. Data from the National Alzheimer's Coordinating Center can be requested using an online request process, outlined here: https://naccdata.org/requesting-data/data-request-process.

## Ethics statement

Ethical approval was not required for this study involving humans in accordance with guidelines on secondary use of existing, public use datasets. Data collected by the ADRCs contributing to NACC were reviewed and approved by their respective IRBs. Written informed consent was obtained from all participants and co-participants in NACC.

## Author contributions

MT: Formal analysis, Investigation, Methodology, Software, Visualization, Validation, Writing – original draft, Writing – review & editing. RS: Formal analysis, Investigation, Methodology, Software, Writing – original draft, Writing – review & editing. CS: Funding acquisition, Resources, Supervision, Validation, Writing – review & editing. YR: Conceptualization, Data curation, Methodology, Project administration, Software, Supervision, Validation, Visualization, Writing – original draft, Writing – review & editing.
