# Supplementary material for: Alzheimer's disease detection using data fusion with a deep supervised encoder
Source: Front Dement. 2024 Feb 12;3:1332928. doi: 10.3389/frdem.2024.1332928 (PMC11271260; doi:10.3389/frdem.2024.1332928)
Supplement: Supplementary file 1 [file Data_Sheet_1.pdf]

Supplementary Material

1 SUPPLEMENTAL METHODS

For the cerebrospinal fluid (CSF) modality, we performed K-nearest-neighbor (KNN) imputation to fill in any missing values, resulting in 1,653 subjects with assay records, each containing three protein biomarker features. This allowed us to retain the most amount of complete patient information by learning from existing data available in the training dataset. We did not include CSF in any multimodal dataset due to very limited sample size when including CSF alongside the other modalities. Additionally, due to the lower dimensionality of the CSF modality, the CSF features were never selected as one of the top features when looking at feature importance in a multimodal dataset. Due to these reasons, we proceeded to only report single-modal results for the CSF dataset.

Out of all the analysis pipelines, only Pipeline c, which we named ”CSF”, involved models using CSF data. As there were only three CSF features, this modality had too few features so no dimensionality reduction was necessary. Thus, we designed only three models for this pipeline, each corresponding to one of the classification methods, including logistic regression for binary classification and random forest and neural network for multiclass classification.

For the AE pipeline, we used the following architecture:

1. Dense layer with ReLU activation with 64 nodes
2. Dense layer with ReLU activation with 32 nodes
3. Dense layer with ReLU activation with 4 nodes (bottleneck layer)
4. Dense layer with ReLU activation with 32 nodes
5. Dense layer with ReLU activation with 64 nodes

The final layer was connected to a dense layer with a softmax activation function to match to the original input features.

For the SE pipeline, we used the following architecture:

1. Dense layer with ReLU activation with 64 nodes
2. Dense layer with ReLU activation with 64 nodes
3. Dense layer with ReLU activation with 10 nodes (latent representation layer)

The final layer was connected to a dense layer with a softmax activate function with three nodes to predict the corresponding diagnosis label in a supervised manner while extracting meaningful latent features.

2 SUPPLEMENTARY TABLES

Results from Pipeline c are reported in Table S1. As expected, the binary classification results are better than the multiclass classification results, with the neural network achieving significantly higher accuracy results than the random forest. In comparison to the other single-modal models (UDS or MRI), the performance of the CSF input models is significantly worse than that of the top UDS single-modal models and significantly better than that of the top MRI single-modal models. This suggests that the CSF features do offer valuable insight for the classification task and should be considered in a large, multimodal dataset in future work.

Table S1. Baseline Models with single-modal CSF input. Test set classification accuracy values are reported for baseline models with single-modal input from the CSF dataset.

	CSF		
	**Binary**	**Multiclass**	
	**Logistic Reg.**	**Random Forest**	**Neural network**
No DR	0.808 (0.802, 0.813)	0.746 (0.741, 0.752)	0.770 (0.764, 0.777)

Table S2. Uniform Data Set (UDS) features from NACC used in the model training process.

UDS Features						
NPSYDEV	NACCAPOE	MMSEORDA	MMSEORLO	PENTAGON	NACCMMSE	LOGIMEM
MEMUNITS	MEMTIME	DIGIF	DIGIFLEN	DIGIB	DIGIBLEN	ANIMALS
VEG	TRAILA	TRAILARR	TRAILALI	TRAILB	TRAILBRR	TRAILBLI
BOSTON	MOCATOTS	DECSUB	BILLS	TAXES	SHOPPING	GAMES
STOVE	MEALPREP	EVENTS	PAYATTN	REMDATES	TRAVEL	NOGDS
SATIS	DROPACT	EMPTY	BORED	SPIRITS	AFRAID	HAPPY
HELPLESS	STAYHOME	MEMPROB	WONDRFUL	WRTHLESS	ENERGY	HOPELESS
BETTER	NACCGDS	NPIQINF	NPIQINFX	DEL	DELSEV	HALL
HALLSEV	AGIT	AGITSEV	DEPD	DEPDSEV	ANX	ANXSEV
ELAT	ELATSEV	APA	APASEV	DISN	DISNSEV	IRR
IRRSEV	MOT	MOTSEV	NITE	NITESEV	APP	APPSEV
FOCLSYM	FOCLSIGN	NACCNREX	NORMEXAM	OCD	HEIGHT	WEIGHT
BPSYS	BPDIAS	HRATE	VISION	VISCORR	VISWCORR	HEARING
HEARAID	HEARWAID	NACCBMI	ANYMEDS	TOBAC30	TOBAC100	SMOKYRS
PACKSPER	QUITSMOK	CVHATT	CVAFIB	CVANGIO	CVBYPASS	CVPACDEF
CVPACE	CVCHF	CVOTHR	CBSTROKE	NACCSTYR	CBTIA	NACCTIYR
SEIZURES	NACCTBI	TBI	TBIBRIEF	TRAUMBRF	TBIEXTEN	TRAUMEXT
TBIWOLOS	TRAUMCHR	TBIYEAR	NCOTHR	DIABETES	DIABTYPE	HYPERTEN
HYPERCHO	B12DEF	THYROID	ARTHRIT	ARTHTYPE	ARTHTYPX	ARTHUPEX
ARTHLOEX	ARTHSPIN	ARTHUNK	INCONTU	INCONTF	APNEA	RBD
INSOMN	OTHSLEEP	OTHSLEEX	ALCOHOL	ABUSOTHR	ABUSX	PTSD
BIPOLAR	SCHIZ	DEP2YRS	DEPOTHR	ANXIETY	INRELY	INCALLS
PSYCDIS	PSYCDISX	NACCAAAS	NACCAANX	NACCAC	NACCACEI	NACCADEP
NACCAHTN	NACCAMD	NACCANGI	NACCAPSY	NACCBETA	NACCCCBS	NACCDBMD
NACCDIUR	NACCEMD	NACCEPMD	NACCHTNC	NACCLIPL	NACCNSD	NACCPDMD
NACCVASD	NACCFAM	NACCMOM	NACCDAD	NACCAM	NACCAMX	NACCAMS
NACCAMSX	NACCFM	NACCFMX	NACCFMS	NACCFMSX	NACCOM	NACCOMX
NACCOMS	NACCOMSX	NACCFADM	NACCFFTD	INEDUC	INRELTO	INKNOWN
INLIVWTH	INVISITS					

Given that we obtained two multiclass classifiers, we also implemented an ensemble classifier using these two models. The ensemble model for multiclass classification using CSF inputs achieved an accuracy value of 76.5% (95% confidence interval: 75.9% to 77.2%) on the test set.

Next, we report all the features used in the model training and evaluation process for each modality of data included in this study. Table S2 includes all features from the Uniform Data Set (UDS) that was used for model training. Table S3 includes all features from the associated MRI scans that was used for model training. Table S4 includes all fluid biomarker features taken from cerebrospinal fluid (CSF) specimens.

Table S3. Magnetic resonance imaging (MRI) volumetric and cortical thickness features from NACC used in the model training process.

MRI Features						
NACCICV	NACCBRNV	NACCWMVL	CSFVOL	GRAYVOL	WHITEVOL	WMHVOL
HIPPOVOL	CEREALL	CERETISS	CERECSF	CEREGR	CEREWH	LHIPPO
RHIPPO	LLATVENT	RLATVENT	LATVENT	THIRVENT	LFRCORT	RFRCORT
FRCORT	LOCCORT	ROCCORT	OCCCORT	LPARCORT	RPARCORT	PARCORT
LTEMPCOR	RTEMPCOR	TEMPCOR	LCAC	LCACM	LCMF	LCMFM
LCUN	LCUNM	LENT	LENTM	LFUS	LFUSM	LINFPAR
LINFPARM	LINFTEMP	LINFTEMM	LINSULA	LINSULAM	LISTHC	LISTHCM
LLATOCC	LLATOCCM	LLATORBF	LLATORBM	LLING	LLINGM	LMEDORBF
LMEDORBM	LMIDTEMP	LMIDTEMM	LPARCEN	LPARCENM	LPARHIP	LPARHIPM
LPARSOP	LPARSOPM	LPARORB	LPARORBM	LPARTRI	LPARTRIM	LPERCAL
LPERCALM	LPOSCEN	LPOSCENM	LPOSCIN	LPOSCINM	LPRECEN	LPRECENM
LPRECUN	LPRECUNM	LROSANC	LROSANCM	LROSMF	LROSMFM	LSUPFR
LSUPFRM	LSUPPAR	LSUPPARM	LSUPTEM	LSUPTEMM	LSUPMAR	LSUPMARM
LTRTEM	LTRTEMM	RCAC	RCACM	RCMF	RCMFM	RCUN
RCUNM	RENT	RENTM	RFUS	RFUSM	RINFPAR	RINFPARM
RINFTEMP	RINFTEMM	RINSULA	RINSULAM	RISTHC	RISTHCM	RLATOCC
RLATOCCM	RLATORBF	RLATORBM	RLING	RLINGM	RMEDORBF	RMEDORBM
RMIDTEMP	RMIDTEMM	RPARCEN	RPARCENM	RPARHIP	RPARHIPM	RPARSOP
RPARSOPM	RPARORB	RPARORBM	RPARTRI	RPARTRIM	RPERCAL	RPERCALM
RPOSCEN	RPOSCENM	RPOSCIN	RPOSCINM	RPRECEN	RPRECENM	RPRECUN
RPRECUNM	RROSANC	RROSANCM	RROSMF	RROSMFM	RSUPFR	RSUPFRM
RSUPPAR	RSUPPARM	RSUPTEM	RSUPTEMM	RSUPMAR	RSUPMARM	RTRTEM
RTRTEMM						

Table S4. Cerebrospinal fluid (CSF) features from NACC used in the model training process.

CSF Features		
CSFABETA	CSFTTAU	CSFPTAU
